# Treatment with, Resveratrol, a SIRT1 Activator, Prevents Zearalenone-Induced Lactic Acid Metabolism Disorder in Rat Sertoli Cells

**DOI:** 10.3390/molecules24132474

**Published:** 2019-07-05

**Authors:** Peirong Cai, Nannan Feng, Wanglong Zheng, Hao Zheng, Hui Zou, Yan Yuan, Xuezhong Liu, Zongping Liu, Jianhong Gu, Jianchun Bian

**Affiliations:** 1 College of Veterinary Medicine, Yangzhou University, Yangzhou 225009, Jiangsu, China; 2 Jiangsu Co-innovation Center for Prevention and Control of Important Animal Infectious Diseases and Zoonoses, Yangzhou 225009, Jiangsu, China; 3 Joint International Research Laboratory of Agriculture and Agri-Product Safety of the Ministry of Education of China, Yangzhou University, Yangzhou 225009, Jiangsu, China

**Keywords:** Sertoli cells, zearalenone, lactic acid, SIRT1, resveratrol

## Abstract

Zearalenone (ZEA) interferes with the function of the male reproductive system, but its molecular mechanism has yet to be completely elucidated. Sertoli cells (SCs) are important in the male reproductive system. Silencing information regulator 1 (SIRT1) is a cell metabolism sensor and resveratrol (RSV) is an activator of SIRT1. In this study we investigated whether SIRT1 is involved in the regulation of ZEA-induced lactate metabolism disorder in SCs. The results showed that the cytotoxicity of ZEA toward SCs increased with increasing ZEA concentration. Moreover, ZEA induced a decrease in the production of lactic acid and pyruvate of SCs and inhibited the expression of glycolytic genes and lactic acid production-related proteins. ZEA also led to a decreased expression of SIRT1 in energy receptors and decreased ATP levels in SCs. However, the ZEA-induced cytotoxicity and decline in lactic acid production in SCs were alleviated by the use of RSV, which is an activator of SIRT1. In summary, ZEA decreased lactic acid production in SCs, while the treatment with an SIRT1 activator, RSV, restored the inhibition of lactic acid production in SCs and reduced cytotoxicity of ZEA toward SCs.

## 1. Introduction

Zearalenone (ZEA) is an estrogen-like mycotoxin that is a common contaminant in cereal crops around the world [1,2]. ZEA is also an endocrine disruptor that is thought to have health effects and global economic impacts [3]. Moreover, ZEA has been shown to be immunotoxic, carcinogenic and genotoxic, as well as to have endocrine inhibiting activities [4]. Because ZEA and its metabolites are similar in structure to 17β-estradiol, they bind to estrogen receptors and trigger a series of estrogen-like effects that can cause serious damage to the reproductive system [5]. Previous studies have reported that exposure to ZEA can lead to reduction of semen quality and affect sperm fertilization capacity [6,7]. It was further discovered that ZEA can destroy the blood testis barrier, inhibit the biosynthesis and secretion of testosterone, and affect the cytoskeleton of SCs [8,9,10]. The serious damage caused by ZEA to the male reproductive system cannot be ignored. Therefore, in this study, we committed to further explore the mechanism of ZEA toxicity on the male reproductive system.

Sertoli cells (SCs), which are also known as germ cell nursing cells, are an essential part of the reproductive system; located in the spermatogenic epithelium, they play an important role in spermatogenesis [11]. The proliferation, differentiation and survival of germ cells depend on lactic acid, amino acids, lipids, metabolic hormones, and vitamins produced by SCs [12,13,14]. Because of the special relationship between SCs and germ cells, energy metabolism in the seminiferous tubules is considered to have unique characteristics [15]. In particular, SCs have a similar form of glucose metabolism to cancer cells, known as the Warburg-like effect, in which lactic acid is produced as a source of energy for germ cells through its own glycolysis [16]. Studies have found that germ cells can only utilize lactic acid as a source of energy [17].

The metabolism of SCs is strictly regulated by hormones; therefore, abnormal interference with hormones or hormonal substances may impact their metabolism [18]. Moreover, studies have shown that endocrine and metabolic diseases can influence the spermatogenic function of male animals by affecting the production of lactic acid in SCs [19], while environmental toxins can also affect spermatogenesis by reducing the production of lactic acid in SCs [20]. Overall, stable energy metabolism of SCs is a prerequisite for normal maintenance of spermatogenic function.

Silencing information regulator 1 (SIRT1) is a NAD^+^-dependent protein deacetylase that regulates major targets through deacetylation and is involved in different biological processes such as inflammation, cellular metabolism and death [21]. Notably, SIRT1 is also a sensor for metabolism and oxidation of nutrient and energy state changes that play an important role in the testes [22,23]. Specifically, SIRT1 plays an important role in spermatogenesis, and its knockout can cause immaturity of SCs and infertility of animals [24,25,26]. Resveratrol (RSV) is a natural, biologically active polyphenolic compound that reduces oxidative damage, inflammation, and apoptosis in cells [27]. Additionally, RSV can, through allosteric interactions, result in increased activity of SIRT 1, thereby increasing the affinity of SIRT 1 for both NAD^+^ and acetylated substrate [28,29].

Although the mechanism of ZEA-induced male reproductive system damage is still unclear, the energy supply of SCs to germ cells is mainly acheived through the secretion of lactic acid. Therefore, we speculate that ZEA may affect male reproductive function by influencing the process of lactic acid production. As an important energy sensor, the relationship between SIRT1 and SC lactate metabolism is also of interest. Previous studies have shown that SIRT1 plays an important role in glucose homeostasis [30], and RSV can activate SIRT1. Therefore, we evaluated the effects of exposure to different ZEA concentrations on the metabolic status of SCs and the regulation of SIRT1 on lactic acid metabolism. The results presented herein provide new descriptions of molecular mechanisms by which ZEA damages the reproductive system.

## 2. Results

### 2.1. ZEA Inhibits Proliferation of SCs

**Figure 1:**
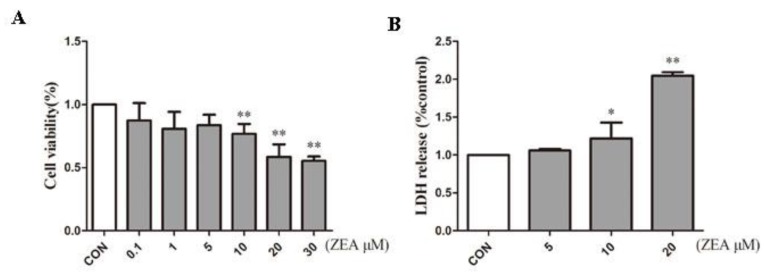
Viability of Sertoli cells (SCs) treated with zearalenone (ZEA). (**A**) SCs in the logarithmic phase were treated with different concentrations of ZEA, after which cell activity was measured using a cck-8 kit. (**B**) ZEA-induced cytotoxicity was detected by lactate dehydrogenase (LDH) release. *p < 0.05, **p < 0.01 versus the control group.

To detect the cytotoxic effects of ZEA on SCs, SCs were treated with ZEA for 24 h. The cell counting kit-8 (CCK-8) assay was used to detect the effects of ZEA on the cell viability of SCs, and the lactate dehydrogenase (LDH) release assay was employed to examine the cytotoxicity of ZEA. As shown in Figure 1A, the viability of the SCs decreased as the ZEA concentration increased. Additionally, when the ZEA concentration was higher than 10 μM, the cells viability decreased significantly. Moreover, the LDH release assay showed that LDH release increased significantly at 10 μM (Figure 1B). According to the previous research in our laboratory, SCs exceeded LD50 when the concentration of ZEA exceeded 30 uM [10]. Therefore, the concentration before 30 uM was selected in this experiment. The results of CCK-8 further confirmed that the concentration we selected was within the LD50. Based on this dosage effect, we selected 0, 5, 10, and 20 μM ZEA as the dose range for subsequent experiments.

### 2.2. ZEA Decreased Lactic Acid Production

**Figure 2:**
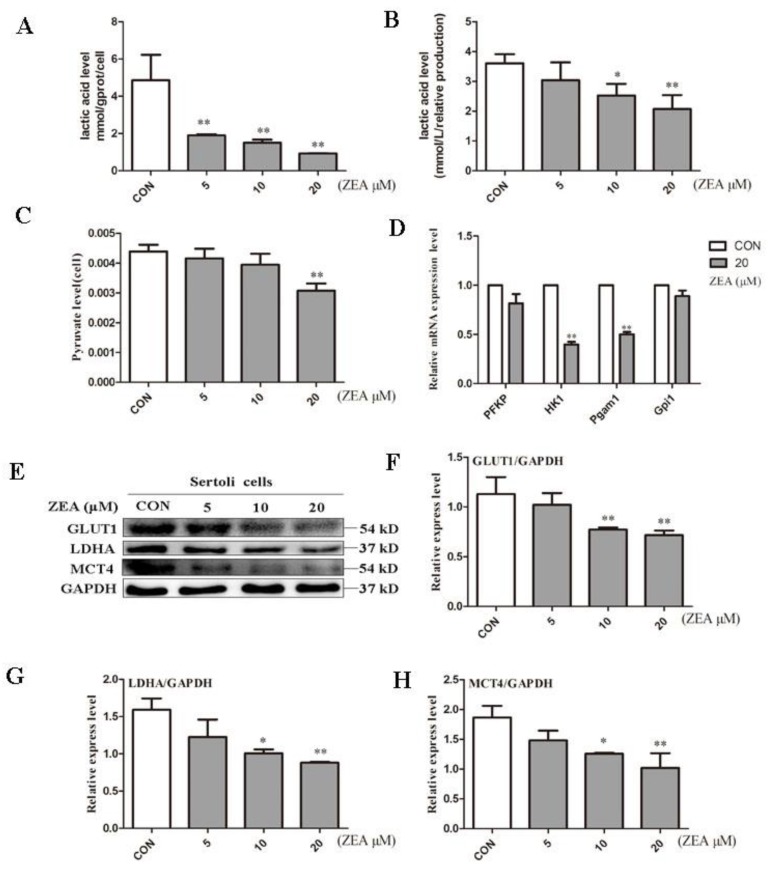
Effect of ZEA on lactic acid production in SCs. (**A**, **B**) A lactic acid kit was used to measure lactate levels inside and outside of cells after exposure to ZEA for 24 h. (**C**) Pyruvate levels in cells after exposure to ZEA for 24 h in SCs detected by the pyruvate kit. (**D**) RT-PCR was used to measure the expression of the key genes PFKP, HK1, Pgam1, and Gpi1 in glycolysis. (**E**) Western blot analysis of the expression of related proteins in the lactate production pathway. (**F**, **G**, **H**) The ratio of glucose transporter 1 (GLUT1)/ glyceraldehyde-3-phosphate dehydrogenase (GAPDH), LDH/GAPDH, and monocarboxylate transporters4 (MCT4)/GAPDH, respectively. *p < 0.05, **p < 0.01 versus the control group.

A lactic acid assay kit was used to measure the lactic acid levels inside and outside the cell. As shown in Figure 2A, the lactic acid levels in the case of 5, 10 and 20 μM were 2.56, 3.24 and 5.22 times lower than the control group, respectively. When the ZEA concentration was higher than 5 μM, the lactic acid produced by the SCs decreased significantly at 10 μM and 20 μM. The lactic acid levels in the case of 10 μM and 20 μM were 1.43 and 2.02 times lower than the control group (Figure 2B). Because of the unique energy metabolism of SCs, most of the pyruvate is converted by LDH to lactic acid. Therefore, we used a pyruvate assay kit to measure the pyruvate level in the cells. As shown in Figure 2C in the case of 20 μM the pyruvate level decreased by 1.43 times when compared with the blank group. To further explore the mechanism of lactic acid production decline, we examined several key genes for glycolysis (Figure 2C). When compared with the control group, we found that the genes HK1 and pgam1 decreased significantly in response to treatment with 20 μM, indicating that ZEA can induce a decrease in glycolysis.

Lactic acid production is a complex process of energy metabolism that is regulated by multiple key proteins. To demonstrate the possible mechanism of lactic acid production decline, we used Western blot analysis to evaluate the key proteins in the lactic acid production pathway. GLUT1, which is a major vector involved in glucose transport in mammals [31], decreased significantly in cells treated with ZEA compared to the control group (Figure 2F). LDH is the key protein responsible for the conversion of pyruvate to lactic acid [32]. Following the production of lactic acid in cells, it can be transported to the seminiferous tubules by MCT4 for use by spermatogenic cells [33]. In the present study, when the concentration of ZEA reached 20 μM, the protein expression of LDH and MCT4 decreased significantly.

### 2.3. ZEA Inhibits Expression of SIRT1 and ATP Level

**Figure 3:**
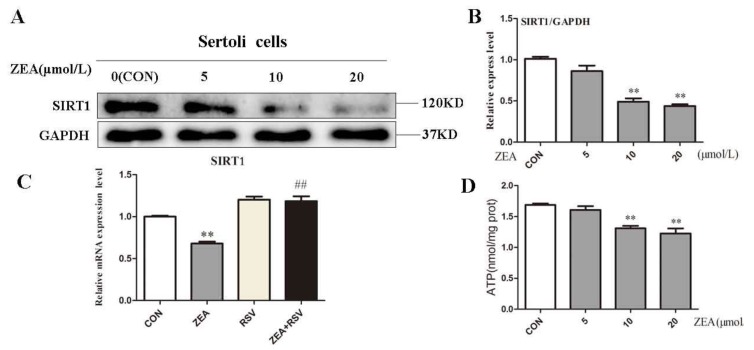
Effects of ZEA and the RSV on the expression of SIRT1 and the intracellular ATP levels. (**A**) Western Blot analysis of the expression of proteins in SIRT1. (**B**) Ratio of SIRT1/GAPDH. (**C**) After SCs were treated with RSV and ZEA for 24 h, RT-PCR analysis of the mRNA expression of SIRT1. (**D**) Effect of ZEA on intracellular ATP levels. *p < 0.05, **p < 0.01 versus the control group, ^##^*p* < 0.01 compared to the 20 μM ZEA group.

Based on the condition of lactic acid metabolism disorders in SCs, we examined the effects of ZEA on the expression of SIRT1 and ATP levels in cells. SCs were treated with different concentrations of ZEA for 24 h. Within a certain range, SIRT1 showed a dose-dependent decrease as the ZEA concentration increased (Figure 3A,B). When compared with the control group, SIRT1 showed a significant decrease in response to treatment with ZEA at 10 μM and 20 μM (Figure 3B). When we added the SIRT1 activator RSV, the SIRT1 gene increased 1.75 times in the co-treatment group compared to the ZEA treatment group (Figure 3C). Intracellular ATP levels also decreased with increasing ZEA concentrations (Figure 3D). These results suggest that ZEA downregulated the expression of SIRT1 and induced SCs energy metabolism disorder, while RSV alleviated the decrease of SIRT1 induced by ZEA.

### 2.4. SIRT1 Activator RSV Treatment Partially Reverses ZEA-Induced Cytotoxicity and the Decline in Lactic Acid Production

**Figure 4:**
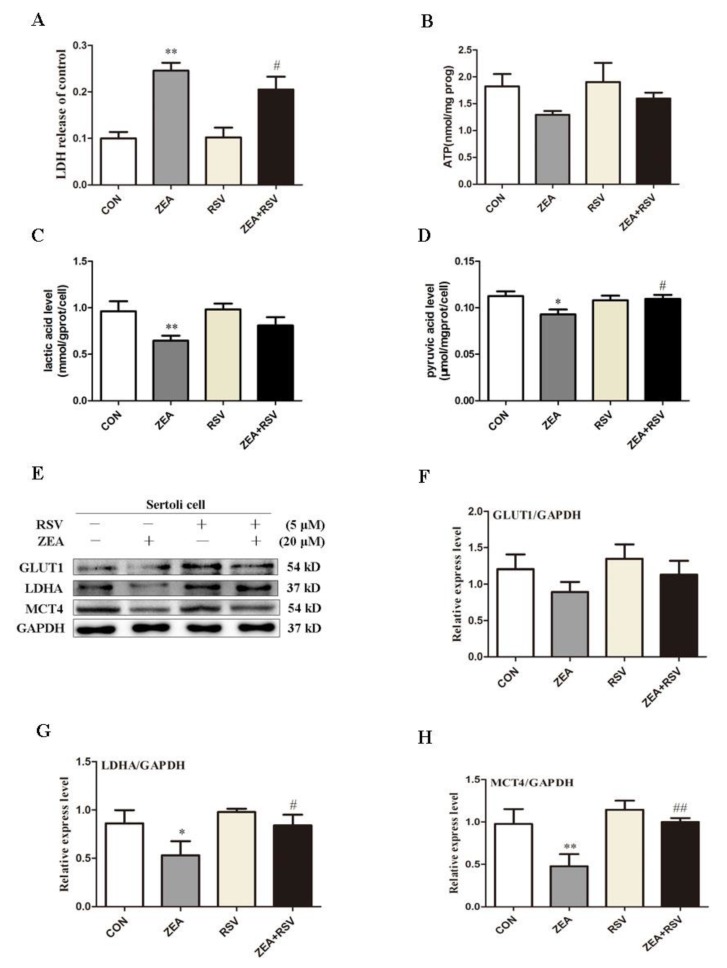
Effects of SIRT1 on ZEA-induced cytotoxicity and lactic acid reduction in SCs. (**A**) After SCs were treated with RSV and ZEA, cytotoxicity was detected using an LDH release kit. (**B**) Following incubation with RSV for 24 h, the effects of ZEA on ATP levels were examined using an ATP kit. (**C**) After SCs were treated with RSV and ZEA for 24 h, the effects of ZEA on intracellular lactate levels were examined by using lactic acid kit. (**D**) SCs were treated with RSV and ZEA for 24 h, and the effects of ZEA on intracellular pyruvate levels were examined by using a pyruvate kit. (**E**) Western blot analysis of the expression of related proteins in the lactate production pathway after incubation with RSV for 24 h. (**F**, **G**, **H**) Ratio of GLUT1/GAPDH, LDH/GAPDH, MCT4/GAPDH. *p < 0.05, **p < 0.01 versus the control group. ^##^*p* < 0.01 compared to the 20 μM ZEA group.

To investigate the effects of ZEA on cell viability after SIRT1 is activated, cells were pretreated with the SIRT1 activator RSV and ZEA for 24 h, after which the release of LDH and intracellular ATP levels were measured. As shown in Figure 4A, LDH release in the ZEA treatment group significantly increased compared to the control group. However, LDH release in the RSV and ZEA co-treatment groups was significantly reduced. In addition, as shown in Figure 4B, co-treatment with RSV and ZEA led to increases in the ATP level relative to the ZEA treatment group. These results indicate that activation of SIRT1 can alleviate ZEA-induced cytotoxicity and energy loss.

To further explore the effects of SIRT1 on the production of lactic acid in SCs, we measured the levels of lactic acid and pyruvate in cells and the expression of relevant proteins generated by lactic acid. As shown in Figure 4C, when compared with the ZEA treatment group, the co-treatment group shown an upward trend in lactate levels. As shown in the Figure 4D, the co-treatment group increased significantly compared with the ZEA treatment group. Moreover, Western blotting showed that LDH and MCT4 decreased significantly in the ZEA treatment group relative to the control group, while GLUT1 decreased, but not significantly (Figure 4F). When compared with the treatment group with ZEA alone, lactic acid production-related proteins increased to varying degrees after adding RSV, with LDH showing a significant increase and MCT4 showing an increase. Taken together, these results indicate that SIRT1 can improve the ZEA-induced decrease in lactate levels.

## 3. Discussion

Mammalian spermatogenesis is a precise periodic and time-controlled process that includes extensive genome and cell remodeling from spermatogonia to haploid cells, and the final release of sperm. This process requires continuous cross-talk between germ cells and SCs. Indeed, SCs are essential for the proliferation and differentiation of spermatogenic cells [34,35]. They provide nutrition, cofactors, and immune exemptions for spermatogenic cells in the seminiferous tubules [36]. Metabolism in the testes is essential for spermatogenesis. The effects of ZEA on endocrine metabolism have also been highlighted, especially damage to the reproductive system [37]. Related studies have shown that androgens, estrogens, melatonin and environmental endocrine disrupting substances can regulate the metabolism of SCs and thus affect the reproductive system [38,39,40]. ZEA and its derivatives play a toxic role by altering steroid metabolism [41,42], which is why we investigated the mechanism of ZEA damage to the reproductive system through SCs.

We observed an increase in the toxicity of ZEA to cells as the concentration of ZEA increased, which was consistent with previous studies in our laboratory [10]. We also explored the effects of ZEA on lactic acid metabolism in SCs and its molecular mechanisms. We found that ZEA reduced the amount of glucose entering the cell by down-regulating the expression of GLUT1, and the level of pyruvic acid in the cells showed a downward trend. However, ZEA down-regulated the expression of LDH, which led to a decrease in pyruvic acid conversion to lactate and a decrease in intracellular lactic acid content. At the same time, the expression of MCT4 was reduced, as was the level of lactic acid transported out of the cells. Spermogenesis is highly dependent on the glucose metabolism of SCs [43], and ZEA can affect the glucose metabolism of SCs. In our study, the lactate metabolism process of SCs generally showed significant changes when ZEA concentration exceeded 5 μM, including the lactate level inside and outside the cell and the expression of lactate-related proteins, which indicated that ZEA less than 5 μM has a relatively small effect on lactate metabolism in cells. This may be related to the special structure of ZEA similar to 17β-estradiol. Zheng et al. have shown that the main toxicity of low dose ZEA is estrogenic effect[44]. It is likely that low dose ZEA mainly causes a series of estrogenic metabolic problems in cells and has little influence on lactate metabolism. It is worth noting that the level of ZEA in cells decreased significantly at 5μM, but the level of extracellular lactic acid showed a downward trend but there was no significant difference. Relevant studies have shown that when cells die, LDH is released as an enzyme with stable enzyme activity, which is also used to detect cytotoxicity. We consider that it is likely that a small portion of pyruvate in the culture medium can also be converted into lactic acid by LDH produced by lysed cells, thus leading to no significant decrease in extracellular lactic acid when intracellular lactic acid decreases significantly. Of course, such results need further verification.

SIRT1 acts as an energy receptor in cells, and their activity is changed by calorie restriction [45]. Conversely, activation of SIRT 1 mimics changes in caloric restriction [46]. These findings indicate a direct link between SIRT1 activity and energy metabolism. The cytotoxicity of ZEA to SCs has been shown to occur via apoptosis, autophagy, and DNA damage [47,48]. In some studies, ZEA has been shown to influence the energy metabolism of SCs, and it can activate the ATP/AMPK signaling pathway through oxidative stress to induce cell cycle arrest [44]. Because of the importance of SIRT1 for energy metabolism in the testes, we linked SIRT1 to lactic acid metabolism. Activation of SIRT1 has been shown to influence SCs proliferation [24]; however, activation of SIRT1 has a protective effect on ZEA-induced Human Embryonic Kidney(HEK) 293 cytotoxicity [49]. When we combined RSV with ZEA on SCs, we observed an increase in lactate levels in SCs and a corresponding decrease in cytotoxicity. In this study, we found that ZEA caused energy loss in SCs and decreased expression of SIRT1. After SIRT1 was activated, the cytotoxicity induced by ZEA was alleviated, and the production of lactic acid showed an upward trend, suggesting that SIRT1 was involved in the regulation of lactate metabolism of SCs. In astrocytes, activation of SIRT1 promotes an increase in lactic acid production [50], and our findings are consistent with those of previous studies.

Lactic acid is not only an energy substrate but also an inhibitor of apoptosis of germ cells [51,52]. Studies have also shown that the addition of lactic acid in vitro can also improve spermatogenesis in the testes [53]. ZEA induces apoptosis in male germ cells [54], except for the toxicity caused by direct action of ZEA and its metabolites on cells, which is probably due to the decrease in lactic acid production by SCs. SIRT1 is a positive regulator of the lactic acid production process of SCs. However, further research is needed to determine whether it can reduce the damage to the reproductive system induced by ZEA.

## 4. Materials and Methods

### 4.1. Reagents Chemicals and Antibodies

Zearalenone (ZEA) was purchased from Sigma–Aldrich (St. Louis, MO, USA); resveratrol (RSV) was purchased from Solarbio (Beijing, China, SR8070); DMEM/F-12 medium was obtained from Gibco (Grand Island, NY, USA, 12500-062); fetal bovine serum (FBS) was obtained from Gemini (California, USA, 900-108). RIPA lysate and protease inhibitor complex were purchased from Pulilai (Beijing, China, C1053); GAPDH and SIRT1 antibody were purchased from Cell Signaling Technology (Boston, Massachusetts, USA); GLUT1, LDH, and MCT4 antibodies were purchased from Abcam (Cambridge, MA, USA); LDH release assay was obtained from Beyotime Institute of Biotechnology (Shanghai, China, C0017); the lactic acid assay kit and the pyruvate assay kit were purchased from Nanjing Institute of Biological Research (Nanjing, China, A019-2, A081); and all other reagents and chemicals were analytical grade and were obtained commercially.

### 4.2. Cell Cultures

All experimental procedures were performed in accordance with the recommendations of the National Research Council Guidelines for the Protection and Use of Experimental Animals and approved by the Animal Care and Use Committee of Yangzhou University (Yangzhou University Medical Center, Approved ID: SYXK (SU) 2017-0044). Male Wistar rats were provided by Yangzhou University Medical Center at 18 to 21 days old. Rats were sacrificed by cervical dislocation, after disinfection of the abdomen, transferred to a clean bench and the abdominal cavity was opened to expose the testicles. After separating the testes we washed them three times with sterile PBS, and then peeled off the tunica albuginea and adipose tissue on the surface of the testes. The testicles were torn using ophthalmic forceps and digested with 0.25% trypsin for 20 min. Subsequently, 5% collagenase was added to continue digestion for 15 min. After the tissue was digested thoroughly, FBS was added to terminate digestion, and the tissue fluid was filtered through a 100 mesh filter and centrifuged at 1800 r/min for 10 min. The supernatant was discarded, suspended in DMEM/F-12 medium and centrifuged again. Abandoned supernatant and DMEM/F-12 medium containing 10% fetal calf serum was added to suspension, and the cells were transferred to a cell flask. The cells were statically cultured for 24 h at 37 °C with 5% CO2 incubator. The cells were treated with Tris-Hcl at pH = 7.4 and cultured in 37 °C with 5% CO2 incubator. We used approximately 30 rats throughout the experiment. SCs were treated with ZEA (0, 5, 10, 20 μM) and RSV (5 μM). The control cells were treated with dimethyl sulfoxide (DMSO).

### 4.3. LDH (Lactate Dehydrogenase) Release Assay for Cytotoxicity

Apoptosis or necrosis of cells causes destruction of the cell membrane structure, resulting in the release of intracellular enzymes into the culture solution, while LDH is relatively stable in enzyme activity. LDH release is considered to be an important indicator of cell integrity and is widely used for cytotoxicity assays; cells are seeded at a density of 5 × 10^3^ cells/well in 96-well plates and allowed to adhere overnight. The cells were then exposed to various concentrations of ZEA and incubated for 24 h. LDH analysis was performed according to the manufacturer’s instructions for the LDH cytotoxicity assay kit (Beyotime, Shanghai, China, C0017). The absorbance of each well was read at 490 nm using an ELx800 Absorbance Microplate Reader (BioTek Instruments, USA).

### 4.4. Lactic Acid Assay Kit to Detect Intracellular and Extracellular Lactate Levels

Cells were cultured at 4 × 10^5^ cells/well in 60 mm dish and allowed to adhere overnight. The cells were then exposed to various concentrations of ZEA and incubated for 24 h. Lactic acid level was performed according to the manufacturer’s instructions for the lactic acid assay kit (Nanjing Jiancheng Bioengineering Institute, Nanjing, China, A019-2). Absorbance detection was performed at 530 nm.

### 4.5. Pyruvate Assay Kit to Detect Intracellular Pyruvate Levels

The pyruvate level was determined using a pyruvate assay kit. In the present study, cells were exposed to various concentrations of ZEA and incubated for 24 h. The pyruvate level was then determined using the pyruvate assay kit according to the manufacturer’s instructions (Nanjing Jiancheng Bioengineering Institute, Nanjing, China, A081). The absorbance was measured at 505 nm.

### 4.6. Western Blotting Analysis

After the cells were exposed to various concentrations of ZEA, the cells were collected using trypsinization. All proteins of the cells were extracted with RIPA lysis buffer. The concentration of the entire protein was detected and adjusted using a BCA protein assay kit. After the whole protein was mixed with the loading buffer and boiled for 10 min, the protein extracted was injected into a sodium dodecyl sulfate polyacrylamide gel, separated according to the size of the protein, and then the protein was transferred to a polyvinylidene fluoride film. The membrane was incubated with 5% skim milk solution; the membrane was then probed with the indicated pro-antibody at a temperature of 4 °C overnight, washed and then incubated with secondary antibody for 2 h at room temperature. Protein signals were detected using an ECL detection system. The polyclonal antibodies against GLUT1 (1:100000, ab115730), LDH (1:1000, ab101562), and MCT4 (1:1000, ab180699) were acquired from Abcam (Cambridge, MA, USA). The polyclonal antibodies against GAPDH (1:1000, 2118) and SIRT1 (1:1000, D1D7) were acquired from Cell Signaling Technology (Boston, MA, USA);

### 4.7. Total RNA Extraction and Quantitative Real-Time PCR

SCs were harvested for extraction of total RNA 24h post-infection by ZEA. According to the manufacturer’s instructions and standard methods, RNA was extracted by using TRIzol (Invitrogen), and convert RNA to cDNA using a reverse transcription kit HiScript Q RT SuperMix for qPCR (Vazyme, Biotech). Each sample was analyzed in triplicate and a reaction mixture with a volume of 20 μL was prepared using ChamQ SYBR qPCR Master Mix (Vazyme, Biotech). The thermal cycling protocol consisted of maintaining the temperature at 95 °C for 30 s, denaturation at 95 °C for 40 cycles for 10 s, and initiating annealing at 60 °C for 30 s for the recycle phase. GAPDH was used to normalize the internal parameters of other mRNA expressions. The primer sequence used for gene are presented in Table 1. According to the mathematical model proposed by Pfaffl, the fold change of target gene expression was calculated using the following formula: 2^-ΔΔCt^.

### 4.8. Statistical Analysis

The results were analyzed using Computer Automated Stowage Planning (CASP) and SPSS22.0 statistical data statistics, and were expressed as mean ± standard deviation (SD). The correlation of different groups was analyzed using one-way ANOVA and LSD multiple comparison analysis. *p* < 0.05 indicates that the difference is significant, and *p* < 0.01 indicates that the difference is extremely remarkable.

## 5. Conclusions

This study revealed the mechanism of ZEA toxicity on SCs from the perspective of the lactic acid metabolism of SCs. ZEA induces decreased lactic acid production by downregulating the expression of glycolytic and lactate-producing pathway-related proteins. Treatment with SIRT1 activator and RSV can also partially alleviate the cytotoxicity and lactic acid production induced by ZEA in SCs.

## Figures and Tables

**Table 1:** Sequence of primers for real-time RT-PCR amplification.

Primers	Primer Sequence	Product Length (bp)
PFKP	Sence:5’- TCAAACTCTCCGAGAACCGTG -3’	160
	Antisense: 5’- TGTCCAAGAATGGTGACCCG -3’	
HK1	Sence: 5’- CATTGTCTCCTGCATCTCCGA -3’	64
	Antisense: 5’- ATTCCGCAATCTAGGCTCGTC -3’	
Pgam1	Sence: 5’- TCGTCATGGCTGCCTACAAG -3’	156
	Antisense:5’-CATAGCCAGCATCTCGCAAC -3’	
Gpi1	Sence: 5’-CCTGTCTACGAACACGGACAA -3’	127
	Antisense: 5’- ATGCAGTGCGATGGAGAGTC -3’	
SIRT1	Sence: 5’- GACGACGAGGGCGAGGAG -3’	363
	Antisense: 5’- ACAGGAGGTTGTCTCGGTAGC -3’	
GAPDH	Sence: 5’- GATGACATCAAGAAGGTGGTGA -3’	1802
	Antisense: 5’- TTATACCGATGTCGTTGTCCCA -3’	

## Abbreviations

BCA	bicinchoninic acid
CCK-8	cell counting kit-8
DMSO	dimethyl sulfoxide
DMEM/F-12	dulbecco’s modified eagle medium: nutrient mixture F-12 (DMEM/F-12)
ECL	electrochemiluminescence
FBS	fetal bovine serum
GAPDH	glyceraldehyde-3-phosphate dehydrogenase
GLUT1	glucose transporter 1
HK1	hexokinase 1
LDH	lactate dehydrogenase
LD50	lethal dose 50
MCT4	monocarboxylate transporters4
M	mol/L
NAD	nicotinamide adenine dinucleotide
PFKP	phosphofructokinase
Pgam1	phosphoglycerate mutase1
PBS	phosphate buffer saline
RSV	Resveratrol
RIPA	radio immunoprecipitation assay
SCs	Sertoli cells
SIRT1	silencing information regulator 1
ZEA	zearalenone.
